# How does gaze to faces support face-to-face interaction? A review and perspective

**DOI:** 10.3758/s13423-020-01715-w

**Published:** 2020-05-04

**Authors:** Roy S. Hessels

**Affiliations:** 1 Experimental Psychology, Helmholtz Institute, Utrecht University, Heidelberglaan 1, 3584CS Utrecht, The Netherlands; 2 Developmental Psychology, Heidelberglaan 1, 3584CS Utrecht, The Netherlands

**Keywords:** Gaze, Faces, Facial features, Social interaction, Dynamic system theory

## Abstract

Gaze—where one looks, how long, and when—plays an essential part in human social behavior. While many aspects of social gaze have been reviewed, there is no comprehensive review or theoretical framework that describes how gaze to faces supports face-to-face interaction. In this review, I address the following questions: (1) When does gaze need to be allocated to a particular region of a face in order to provide the relevant information for successful interaction; (2) How do humans look at other people, and faces in particular, regardless of whether gaze needs to be directed at a particular region to acquire the relevant visual information; (3) How does gaze support the regulation of interaction? The work reviewed spans psychophysical research, observational research, and eye-tracking research in both lab-based and interactive contexts. Based on the literature overview, I sketch a framework for future research based on dynamic systems theory. The framework holds that gaze should be investigated in relation to sub-states of the interaction, encompassing sub-states of the interactors, the content of the interaction as well as the interactive context. The relevant sub-states for understanding gaze in interaction vary over different timescales from microgenesis to ontogenesis and phylogenesis. The framework has important implications for vision science, psychopathology, developmental science, and social robotics.

## Introduction

Understanding how, when, and where gaze or visual attention is allocated in the visual world is an important goal in (vision) science, as it reveals fundamental insights into the organism–environment interaction. Throughout vision science’s history, the dominant approach to attaining this goal has been to study the ‘atomic’ features that ‘constitute’ the visual world—edges, orientations, colors, and so forth (e.g., Marr, 1982)—and determine how they drive the allocation of visual attention and gaze (e.g., Treisman & Gelade, 1980; Itti & Koch, 2000). Humans, as objects in the world that can be looked at or attended, have generally been treated as a special case to the visual system. Yet, in a world so fundamentally social, it would seem that encountering humans is the norm, while encountering single ‘features’—or perhaps a few features combined as in a single red tilted line in the visual field—are the exception.

In this paper, I address the question of how gaze supports, and is an integral part of, social behavior. Specifically, how does gaze to faces and facial features support dyadic face-to-face interactions? I focus on gaze, not visual attention, as gaze can be measured continuously using eye-tracking technology, as opposed to (covert) visual attention which is generally inferred from differences in manual reaction times. Gaze is here defined as the act of directing the eyes toward a location in the visual world, i.e., I thus always consider gaze as being directed somewhere or to something.^1^ Moreover, one’s gaze direction often is accessible to other humans. For example, one can judge where one’s fellow commuter on the train is looking and use this information to either start, or refrain from starting, a conversation. In interaction, gaze can thus support visual information uptake, but also signal information to others.

Previous reviews have addressed the evolution of social gaze and its function (Emery, 2000), how sensitivity to the eyes of others emerges and facilitates social behavior (Grossmann, 2017), the affective effects of eye contact (Hietanen, 2018), and how the neural correlates of gaze (or joint attention in particular) in social interaction can be studied (Pfeiffer et al., 2013), for example through the simulation of social interactions (Caruana et al., 2017). However, there is no review that integrates empirical evidence from multiple research fields on how gaze supports social interaction at the resolution of faces and facial features for (neuro-)cognitive research to build on. Therefore, I introduce a dynamic system approach to interaction in order to understand gaze to faces for the support of social interaction. That this is relevant for vision research stems from the fact that there is a growing appreciation for the hypothesis that the human visual system has evolved for a large part under social constraints, which means that vision may be more ‘social’ in nature than previously considered (Adams et al., 2011).

Apart from the importance for the understanding of social gaze, an integrative theoretical framework of gaze in social interaction has key implications for multiple research fields. First, atypical gaze to people is symptomatic of a number of psychopathologies including autism spectrum disorder (Senju & Johnson, 2009; Guillon et al., 2014) and social anxiety disorder (Horley et al., 2003; Wieser et al., 2009). In both disorders, atypical gaze, such as difficulties in making eye contact, seems particularly evident in interactive settings (as extensively discussed in Hessels et al., (2018a)). A theoretical framework of interactive gaze might shed new light on atypicalities of gaze in these disorders. Second, gaze in interaction is considered an important social learning mechanism for development (e.g., Mundy et al.,, 2007; Brooks and Meltzoff, 2008; Gredebäck et al.,, 2010). Understanding which factors play a role in interactive gaze is a requirement for developmental theories of social learning through gaze. Finally, applied fields such as social robotics may benefit from a model of gaze in interaction to simulate gaze for the improvement of human–robot interaction (see e.g., Raidt et al.,, 2007; Mutlu et al.,, 2009; Skantze et al.,, 2014; Ruhland et al.,, 2015; Bailly et al.,, 2018; Willemse & Wykowska, 2019, for current applications of gaze modeling in virtual agents and social robots).

### Outline of this review

**Fig. 1:**
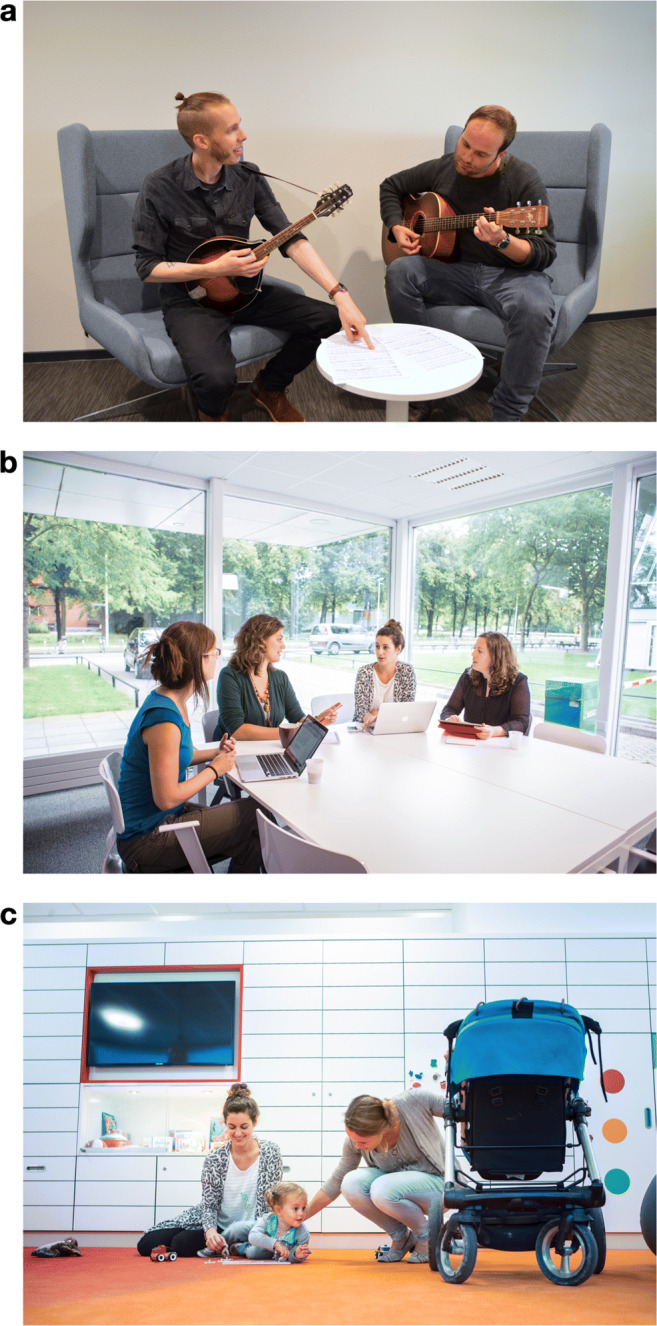
Example face-to-face interactions in which gaze plays an important role. **a** Two musicians learning a song for guitar and mandolin together. Notice how the left person can infer the spatial locus of the right person’s visual attention from his gaze direction. **b** A meeting among co-workers. Gaze direction is often an important regulator of the flow of conversation in such meetings as a key resource for turn allocation. **c** An infant engaged in play with her parent and a third person. Following a parent’s gaze direction is thought to be an important learning mechanism. Picture *a* courtesy of Gijs Holleman, pictures *b* & *c* courtesy of Ivar Pel and the YOUth study at Utrecht University

In order to give the reader a general idea of the framework that I aim to present and of the interactions (see Table 1 for important definitions) to which it applies, consider the following example. In panel A of Fig. 1, two musicians are depicted who are learning to play a song together. Sheet music is placed on the table in front of them. The person on the left seems to be indicating a particular part of the score for the other person to attend, perhaps to point out which chord should be played next. By looking at the eyes of the other, he can verify that his fellow musician is indeed paying attention to the score. Thus, gaze to parts of the face of the other here serves information acquisition about the state of the world. The person on the right clearly needs to look at the score in order to understand which bar the other person is pointing towards. Yet, his gaze direction (towards the table) is observable by the other and may signal to the other where his visual attention is directed. Thus, one’s gaze also affords information, often in combination with head or body orientation. Of course, there is more to social interaction than just gaze. Should the interaction continue, the person on the right might look back to the face of the other and verify whether he has understood correctly that he should play an E minor chord next. From the smile on the left person’s face, he concludes that this is indeed the case.

**Table 1:** Important definitions

Concept	Definition
Stimulus	Content presented to an observer in an experiment, e.g., image or video
Observer	Person observing a set of stimuli
Participant	Person engaged in, or believing to be engaged in or part of, a social situation
Interactor	An agent involved in interaction
Interaction	Reciprocal action or influence between two or more interactors

This example should make it clear that there are at least two important aspects of gaze in face-to-face interaction. On the one hand, visual information is gathered by directing gaze to parts of the visual world. On the other hand, gaze direction may be observable by others, and may thus afford information as well.^2^ The latter is particularly evident in face-to-face meetings including multiple people (such as in panel B of Fig. 1), where gaze can guide the flow of the interaction. Additionally, the fact that gaze may also signal information is thought to be an important aspect of social learning (as in the example depicted in panel C of Fig. 1).

The overarching question of this paper thus is how gaze to faces and facial features supports the face-to-face interactions just described. The following sub-questions can be identified. What visual information is extracted from faces? Does gaze need to be allocated to a particular facial feature to accomplish a given task relevant for interaction, and if so, when? Where do people look when they interact with others? When is gaze allocated to a particular location in the world to acquire visual information, and when to signal information? How is gaze driven by the content of the interaction, e.g., what is said (and done) in interaction? While the goal is to describe how gaze to faces supports face-to-face interaction, much of the relevant research has been conducted in non-interactive situations.

This review proceeds as follows. I first review the evidence in regard to the question when gaze *needs* to be allocated to a particular region of a face in order to ensure successful interaction. This part covers whether and when the visual system is data-limited (cf. Norman and Bobrow, 1975), i.e., when visual information is required in order for successful social interaction to ensue. Second, I review the face-scanning literature to ascertain how humans look at other people, and faces in particular, and whether gaze to faces is dependent on the content of that face, the task being carried out, and the characteristics of the observer and the context. In this part, I ask how humans gaze at other humans *regardless* of whether visual information is required or not. The studies covered in these first two sections mainly cover non-interactive settings, i.e., when the stimulus is not a live person, but a photo or video of a person. Note that for these sections, the default stimuli used are static faces (e.g., photographs). I will mention it explicitly when videos or a live person was used. Third, I review the observational literature on the role of gaze in regulating interaction. Fourth, I review the recent work that has combined eye-tracking technology and the study of interaction proper. Finally, I sketch the overall picture of gaze to faces in support of social interaction and propose a dynamic system approach to gaze in interaction for future research to build on. I end with important outstanding questions for research on this topic.

## Functional constraints of gaze for information acquisition from faces

Humans are foveated animals and use movements of the eyes, specifically saccades, to direct the most sensitive part of the retina (fovea) towards new locations in the visual world. During fixations (i.e., when the same location in the visual world is looked at), objects that appear in the periphery are represented at a lower spatial resolution while objects that appear in central vision (i.e., are projected to the central part of the retina) are represented at a higher spatial resolution. Thus, in order to perceive the visual world in detail, saccades are made continuously, usually at a rate of 3–4 per second to project new areas of the visual world to the fovea (see Hessels et al.,, 2018b, for a discussion on the definitions of fixation and saccades).

Studying gaze thus intuitively reveals something about the information-processing strategy used when interacting with the world (e.g., Hooge & Erkelens, 1999; Land et al.,, 1999; Hayhoe, 2000; Over et al.,, 2007). However, gaze doesn’t necessarily need to be directed at an object in the world in order to perceive it. For example, one need not look at a car directly to notice it coming towards one. In the context of face-to-face interaction, this question can be rephrased as follows: when does a location on the face (e.g., the mouth or eyes) of another need to be fixated in order to acquire the relevant information which could ensure the continuation of a potential interaction? In the remainder of this section, I address this question with regard to (1) facial identity and emotional expression, which I assume are factors relevant to the establishment of interaction, and (2) the perception of speech and (3) the perception of another’s gaze direction, which I assume are important building blocks for many dyadic, triadic, and multiparty interactions. Note that emotional expressions are relevant to the flow of the interaction as well, but in its dynamic nature rather than as a static expression (as they have often been used in eye-tracking research). I return to this point later.

### Facial identity, emotional expressions, and gaze

Facial identity has been an important area of study, particularly with regard to learning and recognizing faces. The consensus in the literature is that the eye region is an important feature for learning face identities. For example, McKelvie (1976) has shown that masking the eyes of a face impairs face learning and recognition more than masking the mouth (see also Goldstein and Mackenberg (1966)). Sekiguchi (2011) has shown that a group that outperforms another in a facial-recognition task using videos of faces looked longer at the eyes and made more transitions between the eyes than the low-performing group. Caldara et al., (2005) furthermore reported that a patient with prosopagnosia (see e.g., Damasio et al.,, 1982) did not use information from the eyes to identify faces.

Eye-tracking studies have further investigated whether fixations to the eyes are necessary for both encoding and recognizing faces. With regard to encoding, Henderson et al., (2005) reported that making saccades during the learning phase yields better recognition performance for faces than restricted viewing (i.e., not making saccades) and Laidlaw and Kingstone (2017) reported that fixations to the eyes were beneficial for facial encoding, whereas covert visual attention was not. With regard to recognition, Peterson and Eckstein (2012) showed that observers, under time restraints of 350 ms, fixate just below the eyes for the recognition of identity, emotion and sex, which was the optimal fixation location according to a Bayesian ideal observer model. This is corroborated by Hills et al., (2011), who showed that cueing the eyes improves facial recognition performance compared to cueing the mouth area and Royer et al., (2018), who showed that face-recognition performance was related to the use of visual information from the eye region. Hsiao and Cottrell (2008) reported that for facial identity recognition two fixations suffice: more fixations do not improve performance. Finally, reduced viewing time during face learning, but not face recognition, has been shown to impede performance (Arizpe et al., 2019).

The study of gaze during the viewing and identification of emotional expressions has likewise yielded crucial insights into the relation between gaze and information acquisition from faces. Buchan et al., (2007), for example, reported that people generally fixate the eyes of videotaped faces more during an emotion-recognition task than during a speech-perception task. However, recognition of emotional expression is often already possible within 50 ms (Neath and Itier, 2014), and does not depend on which feature is fixated (see also Peterson & Eckstein, 2012, and the Section *Face scanning* below). In other words, it seems that the recognition of emotional expressions is not limited by having to fixate a specific region on the face. Indeed, Calvo (2014) have shown that the recognition of emotional expressions in peripheral vision is possible. Performance in peripheral vision is best for happy faces and is hardly impaired by showing only the mouth. However, in face-to- face interaction, it is unlikely that emotional expressions are constantly as pronounced as they are in many studies on the perception of emotional expressions. Emotional expressions in interaction are likely more subtle visually (see e.g., Jack & Schyns, 2015), and can likewise be derived from the context and, for example, speech content, acoustics (Banse & Scherer, 1996), intonation (Bänziger & Scherer, 2005), gaze direction (Kleck, 2005), and bodily movement (de Gelder, 2009). As a case in point, Vaidya et al., (2014) showed that fixation patterns predicted the correct categorization of emotional expressions better for subtle expressions than for extreme expressions. In other words, gaze may be more important for categorizing subtle emotional expressions as they occur in interaction than extreme expressions as often used in emotion-recognition experiments.

### Speech perception and gaze

The perception of speech is one of the building blocks of face-to-face interaction. Although one may assume it is mainly an auditory affair, it has long been known that the availability of visual information from the face increases intelligibility of speech embedded in noise, such as white noise or multi-talker noise (e.g., Sumby and Pollack, 1954; Schwartz et al.,, 2004; Ma et al.,, 2009). The question then is what area of the face is important for the perception of speech, and whether gaze needs to be directed there in order to perceive it. Intuitively, the mouth is the main carrier of visual information relevant to speech perception. However, movement of other facial regions is predictive of vocal-tract movements as well (Yehia et al., 1998). Lansing and McConkie (1999) have further shown that the upper face is more diagnostic for intonation patterns than for decisions about word segments or sentence stress.

With regard to gaze during speech perception, Vatikiotis-Bateson et al., (1998) have shown that the proportion of fixations to the mouth of videotaped faces increased from roughly 35 to 55% as noise (i.e., competing voices and party music) increased in intensity. Moreover, the number of transitions (i.e., saccades between relevant areas in the visual world) between the mouth and the eyes decreased. Buchan et al., (2007) showed that gaze was directed closer to the mouth of videotaped faces during speech perception than during emotion perception, and even closer to the mouth when multi-talker noise was added to the audio. Median fixation durations to the mouth were also longer under noise conditions compared to no-noise conditions. In slight contrast to the findings from Buchan et al. (2007) and Vatikiotis-Bateson (1998), Buchan et al. (2008) showed that the number of fixations to the nose (not the mouth) of videotaped faces increased during speech perception under multi-speaker noise, and the number of fixation to the eyes and mouth decreased. However, fixation durations to the nose and mouth were longer when noise was present, and fixation durations to the eyes were shorter. Yi et al., (2013) showed that when noise was absent, fixating anywhere within 10^∘^ of the mouth of a single videotaped talker was adequate for speech perception (the eye-to-mouth distance was approximately 5^∘^). However, when noise in the audio and a distracting second talking face was presented, observers made many more saccades towards the mouth of the talking face than when noise was absent. Finally, developmental work by Lewkowicz and Hansen-Tift (2012) has shown that infants start looking more at the mouth of videotaped faces around 4-8 months of age, presumably to allow infants to pick up (redundant) audiovisual information for language learning.

A classic example showing that visual information from the face can influence speech perception is the McGurk effect (McGurk & MacDonald, 1976): If an auditive and visual syllable do not concur, a different syllable altogether is perceived. Paré et al., (2003) have shown that this effect diminishes slightly when looking at the hairline compared to the mouth, diminishes substantially when looking 10–20^∘^ away from the talker’s mouth, and is negligible only at 60^∘^ eccentricity (the eye-to-mouth distance was approximately 5^∘^). There is thus substantial influence of visual information from the face, and the mouth area in particular, that affects perception even when looking away from the face. In sum, it seems that the mouth is an important source of information for the perception of speech. Visual information from the mouth can be used for perception even when not looking at the face, although the mouth is looked at more and for longer durations when the conditions make it necessary (e.g., under high levels of ambient noise). When visual information is degraded, the mouth is looked at less again (Wilson et al., 2016).

### Perception of looking direction and gaze

The perception of another’s gaze direction can be considered as a second building block of face-to-face interaction, as it can reveal the locus of another’s spatial attention. In fact, one’s gaze direction can even automatically cue the spatial attention of others. Early studies on the perception of gaze direction have concluded that, under ideal conditions, humans are experts at perceiving one’s looking direction. It has been estimated that humans are sensitive to sub-millimeter displacements of another person’s iris at 1–2 m observer-looker distance with a live looker (Gibson and Pick, 1963; Cline, 1967). Furthermore, this sensitivity to another person’s gaze direction develops early in life (Symons et al., 1998). In a more recent study, Symons et al., (2004) reported that acuity for triadic gaze, i.e., gaze towards an object in between the observer and a live looker, was equally high (with threshold of around 30 s of arc), and is suggested to be limited by the ability to resolve changes in iris shifts of the looker. Yet, under less ideal conditions (e.g., when the looker does not face the observer directly but with a turned head), both the average error and standard deviation of observer judgements increased (Cline, 1967), although only the average error, not the standard deviation increased in Gibson and Pick (1963).

A number of studies have examined how perception of gaze direction relies on information beyond the eyes alone. Estimates of gaze direction have been shown to be biased by, for example, head orientation (Langton et al.,, 2004; Kluttz et al.,, 2009; Wollaston, 1824; Langton, 2000) and other cues (Langton et al.,, 2000). Many studies have since been conducted on the perception of gaze direction (e.g., Gamer & Hecht, 2007; Mareschal et al.,, 2013a, 2013b), and one important conclusion that has been drawn from this work is that people have the tendency to believe that gaze is directed towards them (see also von Cranach & Ellgring, 1973, for a review of early studies on this topic).

One’s gaze direction has also been shown to cue the spatial attention of other’s automatically. The gaze direction of a face depicted in a photo, for example, can result in shorter manual reaction times to targets that appear in the direction of the face’s gaze direction, and longer reaction times to targets appearing in the opposite direction (Friesen & Kingstone, 1998). This effect is known as the ‘gaze cueing’ effect and has been observed from adults to infants as young as 3 months (Hood et al.,, 1998). Although it has been suggested that reflexive cueing was unique to biologically relevant stimuli (e.g., faces and gaze direction), it has since been shown also to occur with non-predictive arrow cues, although this is perhaps subserved by different brain systems (Ristic et al., 2002). Regardless, gaze cueing is considered an important mechanism in social interaction. For in-depth reviews on the topic of gaze cueing, the reader is referred to other work (e.g., Frischen et al.,, 2007; Birmingham & Kingstone, 2009; Shepherd, 2010). For a model of the development of gaze following, see Triesch et al., (2006).

Again, the important question is whether perceiving one’s gaze direction (or the gaze-cueing effect) requires fixation to the eyes. With regard to the perception of looking direction in general, Loomis et al., (2008) have reported that head orientation of a live person can be judged with high accuracy in peripheral vision (up to 90^∘^ eccentricity), when the head changes in orientation. When the head remains in a fixed position, judgements of its orientation were accurate from peripheral vision up to 45^∘^ eccentricity. With regard to the judgement of gaze direction from the eyes alone, these were accurate only within 8^∘^ eccentricity for an 84-cm observer-looker distance. For a 300-cm observer-looker distance, judgements of gaze direction from the eyes alone were accurate only within 4^∘^ eccentricity. To compare, the mean horizontal eccentricity encompassed by the eye region was 1.7^∘^ for the near condition (84-cm inter-person distance), and 0.5^∘^ for the far condition (300-cm inter-person distance). Florey et al., (2015) similarly reported that the perception of a looker’s gaze direction from the periphery depends mostly on head orientation, not eye orientation. They concluded that the poorer resolution in the periphery is not the only cause of this dependence on head orientation, but other effects such as crowding (see e.g., Toet and Levi, 1992) and the expectation of how heads and eyes are oriented likely contribute. Furthermore, Palanica and Itier (2014) reported that discriminating direct from averted gaze within 150 ms is accurate within 3 to 6^∘^ of face eccentricity. To compare, the eye region subtended 2.5^∘^ horizontally by 0.5^∘^ vertically. With regard to the automatic cueing by gaze direction, Yokoyama and Takeda (2019) reported that a 2.3 by 2.3^∘^ schematic face could elicit gaze cueing effects when presented up to 5^∘^ above and below central fixation, but not 7.5^∘^ above or below.

It is important to realize that where one needs to look in order to perceive another’s gaze direction depends on the accuracy with which another’s gaze direction needs to be estimated. The work by Loomis et al., (2008), for example, exemplifies that making a judgement of whether another looks towards or away from oneself with head and eyes rotated is readily possible from peripheral vision. At the other extreme, making a judgement of whether another looks at one’s eyes or mouth might not even be reliable under foveal scrutiny (see e.g., Chen, 2002). Obviously, within these two extremes, another’s gaze direction may be useful in estimating that person’s locus of spatial attention.

### Interim summary

The allocation of gaze to multiple facial features is beneficial for encoding facial identity. However, recognizing facial identity is near-optimal already within two fixations. The region just below the eyes appears optimal for recognizing identity, emotion, and sex. These findings are likely relevant for establishing, not maintaining face-to-face interaction. For the maintenance of face-to-face interaction, the perception of speech and gaze direction are relevant. Gaze to the mouth can aid speech perception when conditions necessitate it (i.e., under high noise). The perception of gaze direction doesn’t likely require gaze to be directed at the eyes, particularly if the orientation of the head co-varies with the gaze direction. However, a direct link between gaze position on a face (i.e. how far it is away from another’s eyes) and the acuity of gaze-direction perception hasn’t been shown. It is expected that an observer’s gaze needs to be directed towards the eyes for more fine-grained judgements of gaze direction of the other. Finally, it seems relevant that future studies investigate data-limitations (i.e., when gaze is necessary to acquire specific visual information) of the kind described here in actual interactive settings.

## Face scanning

In this section, I review the literature with regard to face scanning behavior under less restrained conditions, for example during prolonged viewing of faces or when the observer is free to look around. I aim to review the evidence with regard to the follow questions: (1) what are the biases in gaze to faces and to what degree are these under volitional control, (2) how is gaze to faces dependent on the content of the face, (3) how is gaze to faces dependent on the task posed to the observer, and (4) how is gaze to faces dependent on characteristics of the observer? Note that the studies in this section have mainly been conducted in non-interactive settings. The (fewer) studies on gaze to faces in interaction proper are covered in a later section.

### Biases in gaze to faces

The classic studies by Buswell (1935) and Yarbus (1967) were the first to suggest that people, faces, and eyes are preferentially looked at. This has since been corroborated by many studies (e.g., Birmingham et al.,, 2008a, 2008b, as well as the many studies that follow). Interestingly, it appears that the bias for faces or eyes cannot be predicted by salience (as defined on the basis of stimulus features such as color, intensity and orientation; Itti and Koch (2000)) for faces (Nyström & Holmqvist, 2008) or eyes (Birmingham et al., 2009), but see Shen and Itti (2012) for an example of where salience of videotaped faces does have some predictive value. Amso et al., (2014) reported that salient faces were looked at slightly more often (71%) than non-salient faces (66%), but this difference is marginal (5%) compared to how often faces were looked at when not being salient.

The bias for looking at faces is already present at birth, as infants preferentially track faces compared to e.g., scrambled faces (Goren et al., 1975; Johnson et al., 1991), and preferentially make the first saccade to faces in complex displays (Gliga et al., 2009). The bias for looking at the eyes seems to develop in the first year after birth. Wilcox et al., (2013), for example, reported that 9-month-olds looked more at eyes than 3–4-month-olds for dynamic faces. Frank et al., (2009) further reported that the bias for looking at faces increased between 3 and 9 months of age, whereas gaze of 3-month-olds was best predicted by saliency (see also Leppänen, 2016). Humans are not the only animals with preferences for looking at conspecifics, faces and eyes. Chimpanzees have been shown to preferentially look at bodies and faces (Kano and Tomonaga, 2009), and rhesus monkeys to preferentially look at the eyes in faces (Guo et al., 2003). Chimpanzees, however, appear to gaze at both eyes and mouth and make saccades often between them (Kano & Tomonaga, 2010), more so than humans.

An important question is to what degree the bias for looking at faces is compulsory. In this regard, it has been shown that faces automatically attract attention (I discuss automatic attraction of gaze in the next paragraph) (Langton et al., 2008), although Pereira et al., (2019) state that this isn’t always the case. Automatic attention-attraction by faces can, however, be overcome by top-down control of attention to support the goals of the observer (Bindemann et al., 2007), e.g., to attend something other than faces. Faces have also been shown to retain attention (Bindemann et al., 2005), already for 7-month-old infants (Peltola et al., 2018). Furthermore, the degree to which attention is maintained by faces is modulated by the emotional expression in the faces. For example, fearful faces have been shown to delay attentional disengagement more than neutral, happy and control faces for infants (Peltola et al., 2008; Peltola et al., 2009), and for high-anxious adults (Georgiou et al., 2005). Angry faces additionally maintained attention longer than happy faces and non-faces for 3-year-old children (Leppänen et al., 2018).

Apart from attracting and maintaining visual attention, several studies have also shown that the eyes automatically attract gaze. Laidlaw et al., (2012), for example, showed that when instructed to avoid the eyes, observers could not inhibit some fixations to the eyes. This was, however, possible for the mouth or for the eyes of inverted faces. Similarly, Itier et al., (2007) have reported that eyes always attracted gaze, even when the eye-region was not task-relevant. In another study, it was shown that although faces were preferentially fixated, the time to first fixation on a face was decreased when giving a different task (i.e., spot people as fast as possible; End and Gamer (2019)).

Finally, a left-side bias in looking at faces has been reported in the literature and the use of information from that side in judging e.g., sex (Butler et al., 2005). A similar bias seems to occur in rhesus monkeys and dogs (Guo et al., 2009). Arizpe et al., (2012), have, however, cautioned that this left-side bias may partly be explained by the position of the initial fixation point.

### Content-dependent gaze to faces

#### Gaze to moving faces, talking faces, and faces making eye contact

Apart from general biases and task-dependent gaze to faces, several studies have suggested that gaze to faces depends on what that face is doing, for example, talking, moving, making eye contact, etc.

As noted before, Buchan et al. (2007, 2008) have shown that gaze to videotaped faces is dependent on the intelligibility of speech, with longer fixations to the mouth and nose under noise conditions, shorter fixations to the eyes, and more fixations to the nose. An important question then is whether gaze is also directed more at the mouth when speech occurs and the conditions are favorable (i.e., speech is intelligible). In a free-viewing experiment with videos of faces, Võ et al., (2012) showed that for audible talking faces, fixations occurred equally often to the eyes, nose, and mouth. For muted videos of faces, fewer fixations to the mouth were observed. Võ et al., (2012) go on to show that gaze is dependent on the content and action of the face (audibility, eye contact, movement), with each its own facial region associated. For example, when the talking person in the video made eye contact (i.e., looked straight into the camera), the percentage of fixations to the eyes increased and the percentage of fixations to the mouth decreased. When the face in the video moved, the percentage of fixations to the nose increased. Similarly, Tenenbaum et al., (2013) reported that infants from 6 to 12 months of age (when language production starts to emerge) looked primarily at the mouth of a talking videotaped face (see also Frank et al., (2012)), but that they looked more at the eyes of a smiling face than the eyes of a talking face. Lewkowicz and Hansen-Tift (2012) corroborated that information from the mouth is important for the development of language skills by showing that, for infants aged between 4 and 12 months, the youngest infants (4–6 months) primarily looked at the eyes, while older infants (8–12 months) looked more to the mouth, presumably to pick up (redundant) audiovisual information from the mouth. Importantly, infants aged 10 months fixated the mouth more (relative to the eyes) than the 12-month-olds. This latter ‘shift’ back towards the eyes did not occur for infants that grow up in a bilingual environment, suggesting that they exploit the audiovisual redundancy for learning language for a longer time (Pons et al., 2015). Foulsham et al., (2010) also showed that speech was a good predictor for which videotaped person was being looked at, although it co-depended on the social status of that speaker. i.e., speakers were looked at more often than non-speakers, but speakers with higher social status were looked at more than speakers with lower social status.

There is also contrasting evidence that suggests that the mouth need not always be looked at when speech occurs. While Foulsham et al., (2010) showed that speech was a good predictor of who was being looked at, observers predominantly looked at the eyes of the person. Moreover, Foulsham and Sanderson (2013) showed that this also occurred for videos from which the sound was removed. In another study, Scott et al., (2019) showed observers three videos of an actor carrying out a monologue, manual actions (how to make a cup of tea) and misdirection (a magic trick ‘cups and balls’). They reported that faces were looked at most during monologues, but hands were looked at much more often during manual actions and misdirections in videos portrayed by an actor. Critically, hearing speech increased looking time to the face, but rather the eyes than the mouth. As noted before, however, information for speech recognition need not be confined to the mouth (Lansing and McConkie, 1999; Yehia et al., 1998). Finally, Scott et al., (2019) showed that eye contact by the actor (during manual activity and misdirection in particular) increased observers’ fixation time to the face.

#### Gaze to emotional faces

Multiple studies have investigated how gaze to faces is dependent on the emotional expression contained in the face, particularly for static emotional expressions. Green et al., (2003) asked observers to judge how the person they saw was feeling and showed that inter-fixation distances (saccadic amplitudes) were larger for angry and fearful facial expressions compared to non-threat related facial expressions. Furthermore, more and longer fixations to the facial features (eyes, nose, mouth) occurred for angry and fearful expressions. The authors interpret their findings as a ‘vigilant’ face-scanning style for threat-related expressions. Hunnius et al., (2011) reported that during a free-viewing experiment, dwell times and the percentage of fixations to the inner features (eyes, nose, mouth) were lower for threat-related (anger, fear) emotional expressions for both adults and infants. This was interpreted as a ‘vigilant’ face-scanning style, albeit a different manifestation than that observed by Green et al., (2003). The eyes of threat-related expressions were looked at less compared to happy, sad, and neutral expressions only by the adults, not the infants. In other work, Eisenbarth and Alpers (2011) asked observers to look at faces and judge the emotional expression as positive or negative. They showed that across emotional expressions, the eyes were fixated most often and the longest. Fixations to the mouth were longer for happy expressions compared to sad and fearful expressions, and the eye-to-mouth index (higher values represent more looking at the eyes relative to the mouth) was lowest for happy faces, then angry faces, and then fearful, neutral and sad faces. Bombari et al., (2013) showed that, during an emotion-recognition experiment, the eye region was looked at less for happy expressions, and the mouth looked at more for fearful and happy expressions, compared to angry and sad facial expressions. Finally, Beaudry et al., (2014) reported that the mouth was fixated longer for happy facial expressions than for other expressions, and the eyes and brow region were fixated longer for sad emotional expressions. No other differences were observed between the emotional expressions.

As a potential explanation of the different gaze distributions to emotional expressions, Eisenbarth and Alpers (2011) proposed that regions that are most characteristic of an emotional expression are looked at. If one considers the diagnostic information (see Smith et al.,, 2005) of seven facial expressions (happy, surprised, fearful, angry, disgusted, sad, and neutral), it seems that for the happy expressions this claim holds, although it is less clear for the other emotional expressions. A potential problem with interpreting these studies in terms of information-usage is that either there is no task (i.e., free-viewing, see also Tatler et al., (2011)), or gaze to the face is not the bottleneck for the task. With regard to the latter, it has been shown that emotion recognition can already be done in 50 ms (e.g., Neath and Itier, 2014), so how informative is the gaze about information-usage during prolonged viewing? In contrast to the studies described in the section *Functional constraints of gaze for information acquisition from faces*, here the necessity of gaze location is more difficult to relate to task performance. It may be expected that during prolonged viewing, recognition of the emotional expressions has already been achieved and that gaze is (partly) determined by whatever social consequences an emotion may have. Clearly, describing face-scanning behavior as ‘vigilant’ seems to suggest so. Indeed, Becker and Detweiler-Bedell (2009), showed that when multiple faces were presented in a free-viewing experiment, fearful and angry faces were avoided already from 300 ms after stimulus onset, suggesting that any threat-related information was processed rapidly in peripheral vision and consequently avoided.

Furthermore, the content of a face, such as the emotional expression, during interaction is dynamic and not static as in many of the studies described in this section. Moreover, it is likely more nuanced and tied closely to other aspects of the interaction such as speech (e.g., intonation). Dynamic aspects of emotional expressions can aid their recognition, particularly when the expressions are subtle or when visual information is degraded (e.g., low spatial resolution). For a review on this topic, see Krumhuber et al., (2013). Jack and Schyns, (2015, 2017) have also discussed in-depth that the human face contains a lot of potential information that is transmitted for social communication, and outline how to potentially study the dynamics of it. I am not aware of any studies available at the time of writing that have investigated gaze to the dynamic emotional expressions in e.g., social interaction and how it depends on the diagnostic information for an expression at each point in time. Blais et al., (2017), however, reported that fixation distributions to emotional expressions were different for dynamic as compared to static expressions, with fewer fixations made to the main facial features (i.e., eyes, mouth) for dynamic expressions. However, face stimuli were only presented for 500 ms with the emotional expression unfolding in this time period, yielding only two fixations on average to compare (with the first one likely on the center of the face due to the position of the fixation cross prior to the face).

### Task-related gaze to faces

Already since the work of Yarbus (1967), it has been known that the task given to a person may affect gaze to faces. Since then, gaze has often been interpreted as a means of extracting visual information from the world for the task at hand. Here, I briefly outline the differences in gaze to faces that have been observed for different tasks. Walker-Smith et al., (1977) have shown that during face learning and recognition gaze is confined to the internal features of the face (eyes, nose, mouth). This holds both for when faces are presented sequentially and side-by-side. Similarly, Luria and Strauss (1978) have shown that the eyes, nose, and mouth are looked at most often during face learning and recognition, and Henderson et al., (2005) noted that most time was spent looking at the eyes during face learning. During face recognition, they reported that gaze was more restricted (primarily to the eyes and nose) than during face learning. Williams and Henderson (2007) furthermore reported that the eyes, nose, and mouth were looked at most (and the eyes in particular) during face learning and recognition for both upright and inverted faces.

A common theory from the early days of face-scanning research was the scan path theory (Noton & Stark, 1971), which held that a face that was learned by fixating features in a certain order would be recognized by following that same order. Walker-Smith et al., (1977) have shown that this model does not hold, as scan paths shown during face learning are not repeated during face recognition (see also Henderson et al.,, 2005). Walker-Smith et al., (1977) proposed a model in which the first fixation provides the gestalt of the face. Subsequent fixations to different facial features are used to flesh out the face-percept. In order to compare faces, the same feature must be fixated in both faces.

With regard to other tasks, Nguyen et al., (2009) have shown that the eye region was looked at most when judging age and fatigue. Cheeks were looked at more for the less-tired faces than for the more tired faces. Eyebrows and the glabella were looked at more for the older half of faces compared to the younger half. In a similar study, Kwart et al., (2012) had observers judge the age and attractiveness of faces. They showed that the eyes and nose were looked at most of the time, with very little difference in the distribution of gaze between the two tasks. Buchan et al., (2007) had observers judge either emotion or speech of videotaped faces and found that observers looked more often and longer at the eyes when judging emotion. Finally, Lansing and McConkie (1999) reported that observers looked more often and longer at the upper face when forming judgements about intonation and more at the mid and lower face when forming judgements about sentence stress or segmentation, which mimics the diagnostic information: The upper face was more diagnostic for intonation patterns than for decisions about word segments or sentence stress.

### Observer-dependent gaze to faces

#### Idiosyncratic face-scanning patterns

A particularly interesting observation that was reported by Walker-Smith et al., (1977) in their early work on gaze during face learning and recognition was that their 3 subjects showed very different scan patterns. Recently, a number of studies have corroborated and extended these findings substantially. Peterson and Eckstein (2013), for example, had observers perform a face-identification task under three conditions: (1) free-viewing a 350 ms presented face, (2) free-viewing a 1500 ms presented face, and (3) a fixed fixation location somewhere on the face with the face presented for 200 ms. Observers showed large inter-individual differences in their preferred fixation locations during the free-viewing conditions, the location of which was highly correlated between the 350- and 1500-ms duration conditions. In other words, some observers preferred to fixate the nose while other preferred to fixate the eyes. Interestingly, restricting fixation location to the eyes for ‘nose-lookers’ degraded face-identification performance, whereas restricting fixation location to the nose degraded face-identification performance for the ‘eye-lookers’. Thus, Peterson and Eckstein (2013) concluded that face-scanning patterns are idiosyncratic and reflect observer-specific optimal viewing locations for task performance.

In subsequent work, Mehoudar et al., (2014) have shown that idiosyncratic face-scanning patterns were stable over a period of 18 months and were not predictive of face-recognition performance. Kanan et al., (2015) have additionally shown that observers not only have idiosyncratic face scanning patterns, but also that they have task-specific idiosyncratic face-scanning patterns (e.g., for judging age or for judging attractiveness). Inferring task from a face-scanning pattern was accurate for eye-tracking data from an individual, but not when inferring task based on eye-tracking data from multiple other observers. Arizpe et al., (2017) have further reported that the idiosyncratic face-scanning patterns of multiple observers could be clustered into 4 groups, respectively having a fixation-density peak over the left eye, right eye, nasion, or nose-philtrum-upper lip regions. Face-recognition performance did not differ between the groups and face-scanning patterns were equally distinct for inverted faces. Finally, it seems that idiosyncratic face-scanning patterns are hereditary to a degree. Constantino et al., (2017) have shown that the proportion of time spent looking at the eyes and mouth was correlated by 0.91 between monozygotic twin toddlers, and only by 0.35 for dizygotic twins. Even spatiotemporal characteristics of gaze to faces, such as when saccades were made and in which direction, seemed to have a hereditary component.

#### Sex-dependent gaze to faces

Several studies have indicated that males and females differ in how they look at faces. In early observational work with live people, it has been reported that females tend to look more at an interviewer than males regardless of the sex of the interviewer (Exline et al., 1965). In recent eye-tracking work using videos, Shen and Itti (2012) have reported that fixation durations to faces, bodies and people were longer for male observers than for female observers. Moreover, males were more likely to look at the mouth, and less likely to look at the eyes, than females. Coutrot et al., (2016) corroborated and extended some of these findings. They showed that fixation durations to faces were longer, saccade amplitudes shorter, and overall dispersion smaller for male observers than for female observers. Furthermore, the largest left-side bias was observed for female observers looking at faces of females. Note that these differences are based on a large eye-tracking data set of 405 participants, looking at 40 videos each.

#### Cross-cultural differences in gaze to faces

Cross-cultural differences in face perception and gaze to faces have been a long-standing area of research. Differences between cultures have been observed for gaze during face learning and recognition, emotion discrimination and free-viewing. Blais et al., (2008), for example, have reported that East-Asian (EA) observers looked more at the nose and less at the eyes compared to Western-Caucasian (WC) observers during face learning, face recognition and judgement of race. Furthermore, EA observers were better at recognition of EA faces, and WC observers of WC faces. The authors suggested that not looking at the eyes for the EA observers may be a gaze-avoidant strategy, as eye contact can be considered rude in some EA cultures. Jack et al., (2009) showed that during an emotion-discrimination task, WC observers distributed their fixations across the facial features (eyes, nose, mouth), whereas EA observers focused mostly on the eyes (cf. Blais et al.,, 2008, during face learning and recognition). Furthermore, Jack et al., (2009) reported that EA observers, but not WC observers, exhibited a deficit in categorizing fearful and disgusted facial expressions, perhaps due to the fact that the eyes were mostly fixated, which do not contain diagnostic information for e.g., disgust (Smith et al., 2005). Jack et al., (2009) thus questioned the suggestion by Blais et al., (2008) that EA observers actively avoided looking into the eyes. Moreover, even if EA observers were to look more at the nose than at the eyes (as Blais et al.,, 2008, suggest), it is unlikely that this is a gaze-avoidance strategy, as observers tend not to be able to distinguish whether they’re being looked in the nose or eyes (e.g., Chen, 2002; Gamer et al.,, 2011) and assume they’re being looked at under uncertainty (e.g., Mareschal et al.,, 2013b).

In a study directly aimed at investigating information use by EA and WC observers during face learning and recognition, Caldara et al., (2010) showed observers faces of which a 2, 5 or 8^∘^ gaussian aperture was visible around the fixation point. WC observers fixated the eyes and partially the mouth for all aperture sizes. EA observers, however, fixated the eye region for the 2 and 5^∘^ aperture, and partially the mouth for the 5^∘^ aperture, but fixated mainly the central region of the face (i.e., the nose) for the 8^∘^ aperture. The authors conclude that EA and WC observers rely on the same information for learning and recognizing faces when under visual constraints, but show different biases when no visual constraints are in place. In a particularly comprehensive set of experiments, Or et al., (2015) showed that both Asian and Caucasian observers’ first fixation during a face-identification task were directed, on average, just below the eyes, which has been shown to be optimal in terms of information acquisition for identity, sex and emotion recognition (Peterson and Eckstein, 2012). Fixations were shifted slightly more to the left for Caucasian observers compared to Asian observers, however (approximately 8.1% of the interocular distance). For the remaining fixations during the 1500- and 5000-ms presentation, no substantial differences in fixation patterns between groups were observed. Greater variability was observed within groups than between groups, and a forced-fixation experiment showed that performance was optimal for idiosyncratic preferred fixation locations (see the section *Idiosyncratic face-scanning patterns*).

In a free-viewing experiment, Senju et al., (2013) showed that cross-cultural differences were already evident for young children. Japanese children aged 1–7 years looked more at the eyes and less at the mouth of videotaped faces than British children of the same age. Moreover, Gobel et al., (2017) reported that EA observers only looked more at the nose and less at the eyes than WC observers when the gaze direction of the videotaped talking face being looked at was direct (as if towards the observer), not when the face’s gaze was averted slightly (as if talking to another person). The authors concluded that cross-cultural differences in gaze to faces need to be considered within the interpersonal context in which gaze is measured.

Thus far I have considered only the cross-cultural differences in gaze to faces from the perspective of the observers. However, multiple studies have reported an ‘own-race’ effect, in that higher recognition performance has been observed for observers viewing faces from their own race compared with faces from another race. With regard to how people scan own-race and other-race faces, a number of studies have been conducted. Fu et al., (2012), for example, reported that Chinese observers spent more time looking at the eyes and less time to the nose and mouth of Caucasian faces than of Chinese faces. Wheeler et al., (2011) furthermore reported that older Caucasian infants (within a range of 6 to 10 months of age) looked more at the eyes and less at the mouth of own-race faces than younger infants, whereas this difference was not observed for other-race faces (see also Xiao et al., (2013), for more in-depth findings). Liu et al., (2011) have finally reported that older Asian infants (within a range of 4 to 9 months of age) tended to look less at the internal features (eyes, nose, mouth) for other-race faces than younger infants, which was not observed for own-race faces. Arizpe et al., (2016), however, argued that differences in gaze to own-race and other-race faces are subtle at best, and are dependent on the exact analysis used. When area-of-interest analyses are used, subtle differences emerge, yet these are not found with spatial density maps (a method that does not make a priori specifications of where differences are expected to arise).

### Interim summary

The studies reviewed in this section have revealed the following. When observers are unrestrained in where they can look or for how long they can look, other people are preferentially fixated over objects, faces over bodies and eyes over other facial features. However, exactly where one looks on the face of another is dependent on a multitude of factors. What the face does— e.g., whether it moves, talks, expresses emotion, or looks directly toward the observer—modulates gaze to the face and seems to attract gaze to the information source (e.g., the mouth for speech), although the evidence is not always clear-cut. Furthermore, the task being carried out by the observer affects gaze to the face, although intra-individual differences in task-specific face-scanning patterns are potentially as large as inter-individual differences. Small sex differences in gaze behavior have been observed, as have cross-cultural differences, depending both on the observer and the person observed. Although cross-cultural differences have been observed in children and adults, and across multiple studies, the differences may be only in initial fixations or dependent on the interpersonal context. Finally, and particularly important, face-scanning patterns are highly idiosyncratic, and are, at least in part, under genetic control (i.e., hereditary).

## Social context and the dual function of gaze

The studies described so far have highlighted how gaze is allocated to faces from a purely information-acquisition perspective, or have described general biases. Over the last years, a large number of researchers have argued that traditional laboratory studies of social attention or social gaze (i.e., gaze to people, faces, and so forth) have misrepresented how gaze may operate in ‘real world’ situations (e.g., Smilek et al.,, 2006; Kingstone et al.,, 2008; Kingstone, 2009; Risko et al.,, 2016; Cole et al.,, 2016; Hayward et al.,, 2017). This critique is particularly concerned with the fact that in interactive situations, one’s gaze direction is available to others too, and there may be social consequences to where one looks. The fact that the contrast between the human iris and sclera is large means that it can easily be distinguished from afar, and this high contrast has been suggested to have had a facilitatory effect on the evolution of communicative and cooperative behaviors (Kobayashi and Kohshima, 1997).

What is of particular importance, is that gaze to faces appears to be sensitive to the particular social context (e.g., Risko & Kingstone, 2011; Richardson et al.,, 2012). Foulsham et al., (2010), for example, had participants look at a video of three people making a decision. Not only did the speaker role (i.e., who spoke at what point in time) predict gaze to that person, but participants also tended to look more at the eyes, face and body of people with higher social status than those of lower social status. Similarly, Gobel et al., (2015) reported that gaze to faces depended on the social rank of that person. They reported that the eye-to-mouth ratio of participants was higher when looking at videotaped people of lower social rank, but lower for people of higher social rank when participants believed the other person would look back at them (at a later point in time—their video was said to be recorded and shown later), compared to when participants believed there was no possibility for the other to look back. The authors argued that the inter-personal difference in social rank predicted gaze to facial features (eyes vs. mouth). These two studies show that interpersonal context may affect gaze to faces, and particularly when the other person is (believed to be) live.

In more direct investigations of the effects of the ‘live’ presence of another person, Laidlaw et al., (2011) showed that participants would hardly look at a confederate in a waiting room, while they would often look at a video stream of a confederate placed in a waiting room. The authors argued that the potential for social interaction here led people to avoid looking at the confederate (see also Gregory and Antolin, 2019; Cañigueral & Hamilton, 2019, who report similar findings). In other work, Foulsham et al., (2011) had participants walk around campus wearing an eye tracker, or look at a video of someone walking around campus. While pedestrians were looked at often in both situations, the timing of it showed subtle differences between the video and live conditions. When participants actually walked around campus, other pedestrians were looked at less at a close distance than when watching the video in the lab. Finally, Laidlaw et al., (2016) showed that people on the street tended to look more often at a confederate carrying out a public action (saying hi and waving) than a private action (saying hi on the phone), and concluded that covert visual attention must have been necessary to assess the intention of the confederate, before gaze was either directed to that person or not. These studies show that general biases for looking at other people, faces and eyes do not necessarily generalize to all contexts.

I do not aim to reiterate the ‘lab vs. the real world’ discussion, as this has often been framed, nor the call for interactive paradigms. The interested reader is referred to Kingstone et al., (2008) for a good starting point on this topic. For in-depth comparisons of methodology across different levels of ‘situational complexity’ (i.e., from watching static faces to full-fledged live interaction) see e.g., Risko et al., (2012) and Pfeiffer et al., (2013). My aim is to integrate the available evidence from multiple research fields to tackle the real problem of describing, understanding, and predicting gaze in social face-to-face interactions. The studies covered above make a number of points clear: (1) gaze may be sensitive to many social factors that are not considered from a purely information-acquisition perspective of gaze, but require an information-signaling perspective of gaze, and (2) evidence on gaze in non-interactive settings may not necessarily generalize to interactive settings. The question then beckons how gaze operates in interaction? There are at least two strands of research to help answer this question. First, there is a large observational literature on gaze in interaction. Second, more recent studies—partly in response to the critique on research using static pictures outlined in this paragraph—have used eye trackers to study gaze in interaction. I review these strands of research below.

## Observational studies of gaze in interaction

In stark contrast to the biases reported in the eye-tracking literature for looking at people and faces, many social interactions that occur throughout a day can be characterized by ‘civil inattention’. This phenomenon, described by Goffman (1966) (p. 83-85), often occurs when two strangers meet and consists of a brief exchange of looks, followed by ignoring each other as a form of courtesy (cf. Laidlaw et al., 2011). In other words, people tend not to look at each other in such situations. As an example of this phenomenon, Cary (1978) reported that participants placed in a waiting room almost always gave an initial look to each other. When no initial look took place, it was unlikely that conversation would ensue between the participants. When an additional exchange of looks occurred, conversation was more likely to follow. In social interactions, gaze may thus serve to refrain from, or initiate, conversation. Many early observational studies have subsequently investigated how gaze may regulate interaction, of which I give a brief overview. The observational research described here is characterized by multiple people interacting in real life, while they are observed or recorded. Gaze is then scored in real time or subsequently from the video recordings and carefully annotated, often supplemented with annotations of e.g., speech or gestures.

Probably one of the most important studies on gaze in interaction was conducted by Kendon (1967), who showed that the time spent looking at the face of another during interaction varies heavily (between 28% and over 70%, cf. the section *Idiosyncratic face-scanning patterns*), both during speaking and listening, and that the number of changes of gaze-direction was highly correlated between partners in a dyad. Kendon further showed that gaze was directed more often towards the other at the end of one’s utterance, which was suggested to be to determine which action might be taken next, e.g., to give up the floor or to continue speaking. Gaze also tended to be directed away from the conversational partner when beginning an utterance, which was suggested to be to actively shut out the other and focus on what one wants to say. Some of these findings are summed as follows (p refers to one of the interactants): *“In withdrawing his gaze, p is able to concentrate on the organization of the utterance, and at the same time, by looking away he signals his intention to continue to hold the floor, and thereby forestall any attempt at action from his interlocutor. In looking up, which we have seen that he does briefly at phrase endings, and for a longer time at the ends of his utterances, he can at once check on how his interlocutor is responding to what he is saying, and signal to him that he is looking for some response from him.”* (p. 42).

Allen and Guy (1977) tested the hypothesis of Kendon (1967) that looking away from the other is causally related to reducing mental load, by investigating the relation of looks away from the conversational partner with the content of the speech. They found that when words relating to mental processes (believe, guess, imagine, know, etc.) or judgements (bad, every, good, some, etc.) were spoken, looks away tended to occur more often than without such words. Furthermore, Beattie (1981) had participants either look freely or fixate the interviewer. While continuous looking at the interviewer did not affect speech speed or fluency, more hesitations (‘ehm’) and false starts (starting a sentence and restarting just briefly afterwards) occurred, suggesting that looking at the other indeed interferes with the production of spontaneous speech. This is known as the *cognitive interference hypothesis*.

Observational studies have further shown that gaze depends on e.g., the content of the conversation (i.e., personal or innocuous questions; Exline et al., (1965)), on personality characteristics (Libby and Yaklevich, 1973), on inter-personal intimacy (Argyle & Dean, 1965; Patterson, 1976), and competition versus cooperation between the interlocutors (Foddy, 1978). For example, Foddy (1978) reported that cooperative negotiation resulted in longer bouts of looking at each other than competitive negotiation, although the frequency was the same across both negotiations. The authors suggested that frequency is related to the monitoring/checking function, while length is related to affiliative functions (cf. Jarick and Kingstone, 2015, for more recent work on this topic). Kleinke (1986) summarizes multiple studies on this topic, stating that gaze can be used to exert social control during persuasion or for asserting dominance through prolonged gaze to the face of the other: *“People generally get along better and communicate more effectively when they look at each other. One exception is in bargaining interactions where cooperation can be undermined when gaze is used for expressing dominance and threat”* (p. 84).

As noted, the brief review I give of the observational literature is necessarily non-exhaustive. Most of the early research on gaze and eye contact in social interaction was reviewed by Argyle (e.g. 1972) and particularly Kleinke (1986), the latter organizing the available evidence within the framework of Patterson (1982) on nonverbal exchange. For a detailed overview, the reader is encouraged to read Kleinke’s review. One of the essential points of his work, however, is that *“gaze synchronization and the operation of gaze in turn taking are less reliable than previously believed because they depend on the context and motives of the interactants”* (p. 81), which means that gaze cannot be fully understood as a regulator of interaction without understanding how personal and contextual factors contribute to gaze to faces, as has already been established above for the role of gaze in information acquisition.

As Bavelas et al., (2002) pointed out, the review of Kleinke (1986) was the last major review on observational research on gaze, with few new studies to (re-)define the field afterwards. In the years after 2000, a number of relevant studies have been conducted on this topic, however. For example, in a study on how (non-)verbal communication aids understanding, Clark and Krych (2004) reported that looks to the face of a person giving instructions occurred when a conflict needed to be resolved. Hanna and Brennan (2007) furthermore showed that the gaze direction of someone giving instruction was rapidly used to disambiguate which object was referred to when the instruction could refer to multiple objects. These studies attest to the fact that information from gaze can be rapidly used depending on the contextual needs of the person in interaction.

The field of conversation analysis is another example which has continued to investigate the role of gaze as an important interactional resource. Apart from the role of gaze in the initiation and participation in interaction, and in the regulation of interaction, gaze is also considered to form independent actions in this field: e.g., to appeal for assistance (e.g., Kidwell, 2009). Kidwell (2005), for example, describes how children differentiate different types of looking from their caregiver in order to prolong or change their ongoing behavior. Stivers and Rossano (2010) investigated how responses in conversation are elicited by extensively annotating conversations. They reported that a response was evoked from a conversational partner based on, among others, gaze, interrogative prosody (e.g., rising pitch at the end of a sentence) and lexico-morphosyntax (word- and sentence-formation). Stivers et al., (2009) have furthermore shown that gaze towards another person is a near-universal facilitator (across 9/10 investigated languages) of a speeded response from the conversational partner. For further research on this topic, the reader is referred to Rossano (2013).

### Interim summary

Gaze plays an important role in initiating and regulating interaction. The initiation of conversation tends to be preceded by one’s gaze being directed towards the conversational partner, and the timing of when gaze is directed towards or away from the conversational partner plays an important role in the turn-taking behavior during interaction. Looking toward a conversational partner can be used to give up the turn, whereas looking away can be used to reduce load while thinking about what to say next. Finally, gaze is but one of multiple cues (e.g., prosody) that aid the regulation of interaction.

## Eye tracking in interaction

The observational studies noted above have often been criticized for being subjective in how gaze is coded, whereas eye-tracking has been hailed as the objective counterpart. Early studies have estimated the validity of analyzing gaze in interaction from observation to be around 70–80% for the best recording techniques (Beattie and Bogle, 1982). See also Kleinke (1986) in this regard, who noted that eye and face gaze cannot be reliably and validly distinguished by observational techniques. This is evident in observational research, which is all restricted to whether one looks towards a face or not. Whether one looks at the eyes, nose, or mouth is not reliably established from observation. This is, however, an important distinction with regard to the studies described in the Sections *Functional constraints of gaze for information acquisition from faces* and *Face scanning*, where eyes, nose and mouth are considered as regions that may carry distinctive information useful for ensuring successful interaction. Eye-tracking studies have provided some remedy to these concerns: gaze direction can be objectively measured, although not all eye trackers are good enough to establish gaze to facial features in interactive settings (see e.g., Niehorster et al.,, 2020, for a discussion). Furthermore, eye-tracking in interaction can be quite challenging (e.g., Clark and Gergle, 2011; Brône & Oben, 2018). In this section, I review the eye-tracking studies that have investigated (some aspect of) gaze in face-to-face interaction.

A number of eye-tracking studies in interaction have corroborated reports from the observational literature. For example, Freeth et al., (2013) reported that participants wearing eye-tracking glasses looked less at the face of the interviewer and more to the background when answering questions than when being asked a question. Furthermore, participants looked more at the face of the interviewer when she made eye contact with the participant than when she averted her gaze. Ho et al., (2015) had two participants fitted with wearable eye trackers and had them play games (20 Questions, Heads Up) in which turn-taking behavior occurred. They showed that gaze to the other person preceded the talking of the other (by about 400 ms on average), and gaze was averted when one started talking up to around 700 ms on average after talking started. Holler and Kendrick (2015) furthermore had three people engage in interaction while wearing eye trackers and showed that the unaddressed interactant shifted their gaze from one speaker to the next speaker around (and often prior to) the end of the first speaker’s turn (see also Hirvenkari et al., 2013; Casillas & Frank 2017, for comparable research in non-interactive settings). Broz et al., (2012) showed that the time spent looking at each other (mutual gaze) of a dyad during face-to-face conversation was correlated positively with the combined level of agreeableness and how well the participants knew each other. Finally, Mihoub et al., (2015) showed that gaze to faces in interaction depended on the interpersonal context, i.e., colleagues versus students. These studies combined show that, as has been previously established in the observational literature, gaze is important in regulating turn-taking behavior in interaction, and is related to contextual characteristics (e.g., personality, familiarity, interpersonal context) .

Important innovations in multiple disciplines are beginning to appear. For example, Auer (2018) conducted a study on the role of gaze in regulating triadic conversation and showed that gaze serves both addressee selection and next-speaker selection separately. When speaking, the speaker’s gaze was distributed across both conversational partners, but the speaker’s gaze was directed to one partner specifically at the end of a turn to offer up the floor. The next speaker would then either start their turn, give a small reply to signal the current speaker to continue, or gaze at the third party to hand on the turn. However, it turned out that these contingencies were weak and that speakers could easily self-select as the next speaker by simply starting to talk at the end of a turn without having been ‘offered the floor’. In another study using eye tracking to build on early observational research, Jehoul et al., (2017) investigated the relation between gazes away from a speaker and ‘fillers’ such as “uh” or “um” in dyadic conversation. They showed that one particular filler (“um”) was more associated with looks away from the conversational partner than another filler (“uh”), highlighting the multimodal nature of communication. In recent developmental work, Yu and Smith (2016) showed that infants’ sustained gaze (or sustained overt attention) to an object was prolonged after their parent also looked at that object, implicating joint attention in the development of sustained attention.

Macdonald & Tatler (2013, 2018) have conducted interesting studies on the role of gaze during cooperative behavior, and particularly in relation to instructions. Macdonald and Tatler (2013) had participants wear eye-tracking glasses while building a block model at the guidance of an instructor. When the instructions were ambiguous and gaze cues were available from the instructor to resolve the ambiguity, participants fixated the instructor’s face more than when such gaze cues were not available or when the instructions were unambiguous. Gazing at the face to resolve the ambiguity of instructions predicted increased performance in picking up the right block for the next move. The authors concluded that gaze cues were used only when necessary to disambiguate other information. Macdonald and Tatler (2018), on the other hand, had dyads make a cake together. Half of the dyads were given specific roles (one chef and one gatherer), the other dyads were not. Participants spent very little time looking at each other, but did look at each other often when receiving instructions. When roles were given, moments of looking at each other were longer, and shared gaze (looking at the same object) occurred faster (regardless of who initiated the first look to the object). In another set of studies, Gullberg & Holmqvist (1999, 2006) investigated how gestures (as a nonverbal source of information that may support verbal information and a means for communicating) are fixated in face-to-face communication. One participant was therefore fitted with a wearable eye tracker and engaged in interaction. Gestures were fixated more often when they occurred peripherally compared to centrally and when the speaker fixated the gesture too. Note, however, that gestures were fixated on less than 10% of the cases, while gaze was directed at the face for most of the time. This occurs even in sign language, where gaze is also directed at the face most of the time (> 80%) (Muir & Richardson, 2005; Emmorey et al., 2009). Regardless, these studies combined show that gaze is attuned to the interactive context.

Two eye-tracking studies in interaction have paid particular attention to idiosyncratic scan patterns (see the Section *Idiosyncratic face-scanning patterns*). Peterson et al., (2016) investigated whether idiosyncratic biases also occurred during interaction. First, participants completed a face-identification task in the lab, based on which they were classified as upper looker, middle looker, or lower looker in faces. Participants were then fitted with a wearable eye tracker and had to walk around campus. All fixations were then classified as being on the face or not using a crowdsourced group of raters (using Amazon Mechanical Turk). Similarly, the position of the upper lip (as a central feature in the face) was determined by a crowdsourced group of raters. The relative location of the first fixation on the face (i.e., where it occurred between the eyes and mouth) was highly correlated across the lab- and wearable eye-tracking experiment. This suggests that idiosyncratic face scanning patterns exist for interactive settings as well, not just for looking at static pictures of faces. Similarly, Rogers et al., (2018) had dyads engage in conversation while wearing eye-tracking glasses. They reported large inter-individual differences in whether the eyes, nose, or mouth were preferentially looked at.

Recently, a series of studies on gaze to facial features during face-to-face interaction has been conducted by Hessels et al. (2017, 2018a, 2019). Hessels et al., (2017) used a video-based interaction setup with half-silvered mirrors that allows one to both look directly into an invisible camera and at the eyes of the other, while their eye movements are recorded with remote eye trackers. They had dyads look at each other for 5 min and reported that participants spent most of the time looking at each other’s eyes, followed by the nose and mouth. Interestingly, the time spent looking at each other’s eyes was highly correlated across dyads (cf. Kendon, 1967, who reports a similar correlation for looking at the face across dyads). In a second experiment, a confederate either stared into the eyes of the other or looked around the face, although this did not affect the gaze of the other participant. Using the same setup, Hessels et al., (2018a) showed that looking at the eyes was correlated to traits of social anxiety and autism spectrum disorder in a student population. Moreover, paired gaze states (e.g., ‘eye contact’ or one-way averted gaze) were highly, but differentially, correlated to social anxiety and autistic traits. Higher combined traits of social anxiety predicted shorter periods of two-way and one-way eye gaze, and a higher frequency of one-way eye gaze (corroborating a hyper-vigilant scanning style). Higher combined autistic traits, on the other hand, predicted a shorter total time in two-way, but a longer total time in one-way eye gaze (corroborating a gaze avoidance scanning style). See Vabalas and Freeth (2016), however, who find no relation between social anxiety or autistic traits and distribution of gaze to the face in a student sample in a wearable eye-tracking interview setting. Finally, Hessels et al., (2019) reported that the eyes, nose and mouth of a confederate were fixated more often and for longer total durations when the participant was listening than while speaking and that this did not depend on whether the confederate himself was looking away or towards the participant. Interestingly, a gaze shift toward and away from the participant by the confederate caused a difference in the distribution of gaze over the facial features of the participants, which was found not to be due to stimulus factors in a second experiment. The authors concluded that the confederate’s gaze shift away from the participant acted as a gaze guide, whereas the gaze shift toward the participant caused participants to distribute their gaze more over the facial features, in relation to the participant’s subtask of monitoring when to start speaking. I.e., a gaze shift away from the participant by the confederate likely meant that the participant didn’t need to start speaking, whereas a gaze shift towards the participant might have signaled this.

### Interim summary

Eye-tracking studies of gaze in interaction have corroborated findings from both the face-scanning literature and the observational literature. Findings that corroborate the face-scanning literature include the bias for looking at the eyes when one looks at the face of another and idiosyncratic face-scanning patterns. Findings that corroborate the observational literature include the relation between looking toward or away from the conversational partner and the production of speech, as well as patterns of gaze at turn start and end, and the relation to personality or interpersonal context. Several eye-tracking studies have also provided critical extensions, which include the finding that a gaze shift may guide another person’s gaze related to the task of monitoring when to start speaking, as well as the rapid use of gaze cues during cooperative behaviors, and the relation between joint gaze to an object and attentional development.

## A perspective

In the Section *Functional constraints of gaze for information acquisition from faces*, I have identified when gaze may need to be directed at specific areas of another’s face for acquiring the relevant information (e.g., speech, gaze direction) in order to ensure successful interaction. In the Section *Face scanning*, I have identified the biases in gaze to faces and how they are modulated by the content of the face and observer-characteristics. In the sections *Observational studies of gaze in interaction* & *Eye tracking in interaction*, I have identified how gaze to faces may regulate social interaction. The studies reviewed here stem from different disciplines and different methodological backgrounds (psychophysical research, observational research, eye-tracking research) with various topics of research (emotion, conversation, interpersonal synchrony, social interaction, etc.). In what follows, I sketch a perspective in order to guide future research on the topic of gaze to faces in social interaction. The goals of this final section are (1) to summarize and organize the relevant factors that might predict gaze to faces in social interaction, (2) to facilitate the development of future studies on this topic across the breadth of the disciplines involved, and (3) to suggest a way how future studies might describe their findings on gaze in the context of multimodal interaction. It should be noted up front that most studies described above have been designed to maximize the effect of one parameter of interest (e.g., task, context, facial expression) on gaze to faces. In a way, researchers have been working on the ‘atomic’ features of social interaction that might drive gaze. An important question is how conclusions from these studies generalize to the complexity of face-to-face interaction and its situational variance. For example, studies on gaze to emotional faces have mostly featured static pictures with prototypical expressions. Yet, in interaction, emotional expressions are likely much more nuanced. They are not static images, but moving faces bound to bodies that likely carry multiple redundant sources of information (intonation, body posture, etc.). In interaction, this *“varied bouquet of ... cues”* (cf. Koenderink et al.,, 2000, p. 69) is available to the observer (or better: interactor). It has been well established that the world is full of redundancy for humans to exploit in guiding their behavior (e.g., Brunswik, 1955).

I propose that one method that may be particularly helpful in guiding future research on gaze in face-to-face interaction is dynamic systems theory (see e.g., Smith and Thelen, 2003), which, as Beer (2000) explains in the context of cognitive science, focuses on how a process or behavior unfolds over time and how the unfolding is shaped by various influences. This approach contrasts with, for example, a computational perspective which might focus on how behavior is causally determined by a set of information-processing mechanisms—i.e., a linear A causes B approach with a set of computations in between. A dynamical approach to (aspects of) human interaction is not new per se. Similar approaches have been proposed and utilized, particularly in research on alignment and synchrony in interpersonal interaction and conversations (see e.g., Fusaroli and Tylén, 2012; Dale et al.,, 2013; Paxton & Dale, 2013; Fusaroli & Tylén, 2016). Such approaches have, however, not been commonly suggested or utilized in e.g., psychophysical research on the role of gaze to faces. However, the tenets of a dynamic system approach can be applied to many aspects of this multidisciplinary research topic. In line with what previous researchers have suggested, a dynamic system approach seems to me particularly suited for the study of social interactions, as interactions unfold over time and stimulus and response are hard to disentangle. An analogy to acoustic resonance might help clarify this latter point. I assume that the reader is familiar with the phenomenon of audio feedback, which, for example, occurs when a microphone and speakers are not configured well, resulting in a continuous sound (often loud and high-pitched). While one can describe how this sound develops over time, claiming the microphone or speaker to be the single cause is illogical. It depends on the configuration of the entire system. In social interaction, an analogous phenomenon may occur when two people are forced to stare at each other. At some point they may burst out in laughter. Now, what is the cause of this outburst? Perhaps it begins with a slight twitch of one’s corner of the mouth, yet a sequence of causes and effects leading up to the laughter can hardly be ascertained. Thus, the emphasis of the present framework is on changes over time in aspects of the interaction and relations between them.

### A dynamic system approach to gaze in face-to-face interaction

**Fig. 2:**
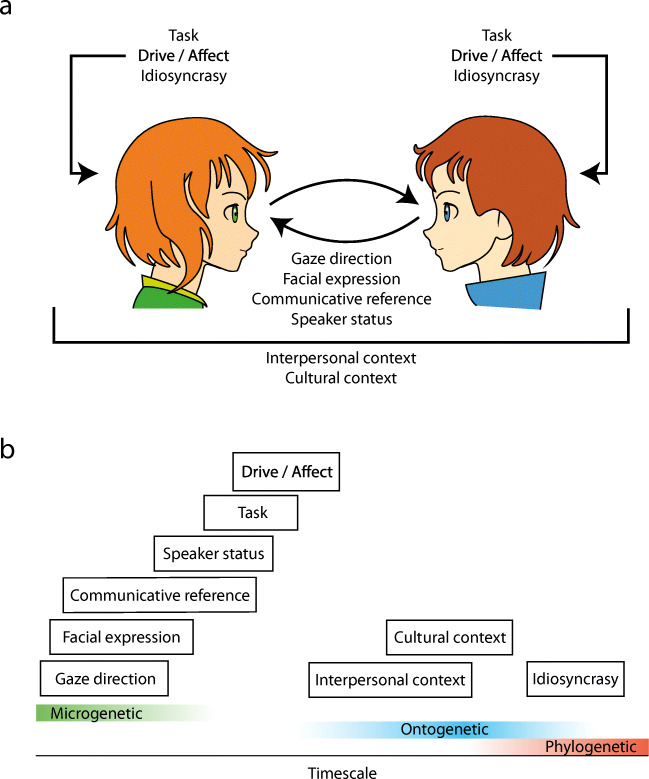
Overview of the framework. **a** Schematic drawing of the relevant aspects of face-to-face interactions considered as sub-states of a dynamic system of interaction. These sub-states are organized as those belonging to the individual interactors (task, drive and affect, idiosyncratic scan patterns), the content of the interaction itself (gaze direction, facial expression, communicative reference, speaker status) and the broader context in which the interaction takes place (interpersonal context, cultural context). **b** Overview of the timescale at which the sub-states of the dynamic system that describes face-to-face interaction are expected to vary, from microgenetic, to ontogenetic and phylogenetic timescales

I propose that face-to-face interaction can be considered as a dynamic system with multiple sub-states that vary at different timescales and that gaze can be considered as one of these time-varying sub-states. I hold that the state of the entire interaction can be characterized by a number of interactor-specific sub-states (task, drive or affect, idiosyncrasy), sub-states relevant to the content of the interaction itself (gaze direction, facial expression, communicative reference, speaker status), and sub-states relevant to the context in which interaction occurs (cultural and interpersonal context). A schematic overview of these sub-states is given in Fig. 2, panel A. It is important to note that, in this framework, the interaction is not considered as two dynamic systems (i.e., the interactors), but one dynamic system which includes two interactors, a context in which the interaction takes place, and the content of the interaction itself (cf. De Jaegher et al.,, 2010; Dale & Spivey, 2018, for similar perspectives on interaction).

In order to grasp how gaze direction is related to the state of the interaction, it is necessary to understand the time-varying characteristics of the other sub-states and at what timescale these sub-states vary. Panel B in Fig. 2 depicts how the sub-states differ in the timescale at which they vary, across microgenetic time (i.e., the timescale at which awareness arises), ontogenetic time (or developmental time) and phylogenetic time (or evolutionary time). At the shortest timescale, facial expressions such as emotional expressions vary as well as facial movements that correlate with speech patterns. One’s gaze direction may (1) correlate with one’s own facial expressions and (2) correlate with another’s facial expressions, such that one’s gaze is directed to the facial features (eyes, nose, mouth, eyebrows, etc.) of another in a predictable fashion based on that person’s facial expression. At a slightly longer timescale, what I term ‘communicative reference’ may vary (e.g., Stukenbrock, 2018). These are, for example, gaze cues or gestures (or both, see Yu & Smith, 2013) which may predict when gaze is directed towards relevant objects, i.e., those that coincide with an interactor’s locus of spatial attention, or those that are the topic of conversation. Speaker status then refers to which of the interactors is speaking and varies with the turn-taking behavior of the interaction. Importantly, gaze direction may be correlated not only to who speaks at what point in time, but also to the time since changes of turn, the time to upcoming turn changes, and the stage of speaking (e.g., thinking of what to say versus saying it) (Bailly et al., 2010). Note that ‘speaker status’ does not suggest that one of the interactors is active, while the other is passive. Both can be actively engaged in the interaction and acquire information from or signal information to each other. It has merely been termed ‘speaker status’ to indicate whether one is primarily speaking or not, which has been shown to predict where one might look (see above).

A particularly relevant aspect of the interaction to consider is the task carried out by an interactor. As has been pointed out in previous research (e.g., Land & Furneaux, 1997; Land et al.,, 1999; Hayhoe, 2000), task is an important predictor of gaze in most daily settings, for example during driving, making tea or sandwiches, reading music or playing sports. As Macdonald and Tatler (2013, 2018) and Hessels et al., (2019) have shown, task is an important predictor of gaze during social interaction as well. At a slightly longer timescale still, drive and affect are expected to vary. This is a rather broad category that relates to long-term emotional states (moods), personality characteristics and e.g., drives to establish, guide, maintain, or dominate a particular interaction. In other words, they are behavioral predictors of gaze that are not task- or context-specific by definition. At the ontogenetic timescale sub-states belonging to the context in which interaction occurs are expected to vary: the interpersonal and cultural context. Finally, idiosyncratic face-scanning patterns are expected to vary at the phylogenetic timescale, and have been suggested to operate as a form of biological niche construction (Constantino et al., 2017).

According to this framework, gaze behavior of two interactors in face-to-face interaction should be investigated as a dynamic phenomenon developing over multiple timescales and investigated in relation to the time-varying characteristics of the other sub-states that compose the entire state of the interaction. One working hypothesis is that sub-states of the interaction generally correlate with gaze to faces and facial features at shorter timescales than personal and contextual sub-states which correlate with gaze to faces and facial features over longer timescales including ontogenesis and phylogenesis.

There are at least three important implications of the framework I propose for current research on the role of gaze in face-to-face interactions:

1. Perhaps self-evident, it is paramount that gaze is investigated in actual interactive contexts.
2. Gaze should not be treated as an isolated phenomenon, but as one aspect of the interaction, which is multimodal by nature.
3. The time-dependency of gaze to faces in interaction should be emphasized, by relating gaze to the time-varying characteristics of the other sub-states of the interaction. This is in contrast to the dominant approach of reporting aggregate measures of gaze averaged over large parts of an interaction.

Note that the sub-states that compose the state of the interaction as described here should not be considered as logical solids. These sub-states are mere descriptors based on the literature reviewed above, but may be more fluid than initially described. For example, the boundary between ‘task’ and ‘drive’ may not be clear-cut, and changes in task may change one’s drive and vice versa. Note also that there is no reference in the framework to the medium through which the interaction takes place and the quality of the information thus transmitted (e.g., a noisy communication channel). Although it may be an important aspect to consider for e.g., video-mediated interactions, I assume the quality of the information is not generally problematic in live interaction, nor is it a characteristic of the interactors, interactional context, or content of the interaction proper.

A criticism one might raise is why the emphasis should be on gaze, as the state of the interaction is composed of many sub-states, of which gaze is but one. As noted in the Introduction, there has been a lot of emphasis on the importance of gaze in social behavior in the literature (e.g., Emery, 2000; Grossmann, 2017; Pfeiffer et al.,, 2013), which was the starting point for the present review. Within the framework here proposed, gaze is considered to be one of multiple sub-states, not necessarily more important than the rest. As such, gaze is perhaps considered to be less ’fundamental’ to social interaction than initially conceived at the start of the review. One advantage of gaze, however, is that it can be measured with a high signal-to-noise ratio using state-of-the-art eye trackers, which makes it an attractive aspect of the interaction to measure. This does not hold for all other aspects of the interaction, such as for example facial expressions, which are difficult to measure reliably using automated techniques (see e.g., Baltrušaitis et al.,, 2015).

#### Towards application of the framework

How might one design new experiments on the role of gaze in face-to-face interaction based on the proposed framework? While investigating gaze within the context of the entire multimodal interaction is theoretically attractive, it might not be practically feasible. To make the framework empirically tractable, one will have to make simplifying assumptions depending on the specific research question.

Say that one is interested in the relation between gaze, facial expression, and speaker status. One could engage dyads into a conversation and operationalize the sub-states of the interaction as follows: Gaze is operationalized for each interactor using an area-of-interest method and is assigned one of the following labels: partner eyes, partner mouth, partner body, partner hands, away from partner. Facial expression is annotated manually for each interactor from a video recording and can take the following states: neutral, laughing, frowning. Speaker status is determined from the audio recording and takes the following states: interactor 1 speaks, interactor 2 speaks, both speak, no one speaks. One may assume that the conversation is too brief for the task, interpersonal context or cultural context sub-states of the interaction to change meaningfully (although the states itself may be important to conceptualize and consider). From here, it is possible to cross-correlate the changes in sub-states of the interaction over time, or to investigate the transition probabilities across combined speaker-expression-gaze states (i.e., as a simplified state of the interaction). In a similar vein, one might be interested in the relation between gaze and the interpersonal context between e.g., a parent and her child. Obviously, the timescale at which one describes changes in the gaze sub-state with respect to changes in the interpersonal context is different from that of the previous example, but the manner of description and analysis may be quite similar. Based on such analyses, one may begin to uncover how gaze supports face-to-face interaction from moment-to-moment, how gaze may stand in for other ostensive signals, or which patterns of gaze are typically elicited in certain interactive contexts.

## Concluding remarks & outstanding questions

In this paper, I have reviewed the literature on how gaze can support social interaction, and in particular dyadic face-to-face interactions. I briefly summarize the conclusions of the review and outline a number of fruitful avenues for future research.

Maintaining face-to-face interaction builds on, among others, the perception of speech and gaze direction. Gaze to the mouth can aid speech perception, while gaze to the eyes likely aids the perception of gaze direction for fine-grained, but not crude judgements of gaze direction. When participants are unrestrained in where they can look, or for how long they can look, there is a bias for fixating other people, faces and eyes. Gaze to faces is, however, modulated by what the face does (talking, expressing emotion, making eye contact), and seems to attract gaze to the source of information. The participant’s task furthermore affects gaze to faces, although intra-individual differences in task-specific face-scanning patterns are large. Face-scanning patterns are further sex- and culturally dependent, highly idiosyncratic, and partly under genetic control.

Gaze plays an important role in initiating and regulating interaction, for example in initiating conversation and turn-taking behavior. Giving up one’s turn often includes a look towards the conversational partner, whereas load may be reduced by looking away. Finally, gaze seems to be tightly interwoven with other cues such as linguistic cues for the regulation of interaction. A substantial proportion of eye-tracking studies in interaction have corroborated observational findings on the initiation and regulation of interaction, as well as idiosyncratic face-scanning patterns from the non-interactive literature. These findings from non-interactive settings thus generalize to interactive situations. More recent eye-tracking studies have begun providing critical extensions of the observational literature.

I have sketched a dynamic system approach to interaction which may provide the basis for future research on the role of gaze in face-to-face interaction. Gaze can thus be investigated in relation to sub-states of the interaction, encompassing aspects of the interactors, the content of the interaction itself as well as the interactive context, with each sub-state varying over different timescales from microgenesis to ontogenesis and phylogenesis. A number of important questions for future research are as follows:

1. Not all evidence comes from interaction proper. Do all findings of gaze to faces hold for interactive situations as well? If so, what is their relative contribution? An essential factor in making these questions tractable is that eye-tracking setups capable of producing high spatial and temporal resolution eye-tracking data are used.
2. The dynamic system approach I propose is merely a framework at present. What would be minimally needed to predict gaze in interaction given the other sub-states of the interaction? Computational approaches may be particular useful in this regard.
3. How do idiosyncratic gaze patterns come to develop? Are they mainly under genetic control (Constantino et al., 2017), or are they modulated through interactions with other people?
4. How are tasks or drives represented for the control of gaze in interaction? When does a task become a drive or vice versa? How wide or narrow are representations of tasks or drives (cf. *“suchbild”* in Koenderink, 2014)?

## Open practices statement

Data sharing not applicable to this article as no datasets were generated or analyzed during the current study. The article was not pre-registered. A preprint has been submitted to: https://osf.io/8zta5.

## Footnotes

1 It is important to realize that one may have the feeling of staring ‘into nothingness’, yet this act may be perceived as a strong social signal by someone else.

2 This fact has been well known for a long time. For example, Kendon (1967) writes: “we shall offer some suggestions as to the function of gaze-direction, both as an act of perception by which one interactant can monitor the behavior of the other, and as an expressive sign and regulatory signal by which he may influence the behavior of the other.” (p. 24). In recent eye-tracking research, the use of photos and videos of faces has been predominant. In this part of the literature, the regulatory signal of gaze-direction may have been, perhaps, overlooked.
